# Individual and familial factors associated with caries and gingivitis among adolescents resident in a semi-urban community in South-Western Nigeria

**DOI:** 10.1186/s12903-021-01527-x

**Published:** 2021-03-27

**Authors:** Morenike Oluwatoyin Folayan, Maha El Tantawi, Nneka Maureen Chukwumah, Michael Alade, Olakunle Oginni, Boladale Mapayi, Olaniyi Arowolo, Nadia A. Sam-Agudu

**Affiliations:** 1 Faculty of Dentistry, Obafemi Awolowo University, Ile-Ife, Osun State Nigeria; 2 Faculty of Dentistry, Alexandria University, Alexandria, Egypt; 3 School of Dentistry, University of Benin, Benin, Edo State Nigeria; 4 Obafemi Awolowo University Teaching Hospitals Complex, Ile-Ife, Nigeria; 5 Department of Mental Health, Obafemi Awolowo University, Ile-Ife, Osun State Nigeria; 6 International Research Center of Excellence, Institute of Human Virology Nigeria, Abuja, Federal Capital Territory Nigeria; 7 Institute of Human Virology and Department of Pediatrics, University of Maryland School of Medicine, Baltimore, USA

**Keywords:** Caries, Gingivitis, Oral health, Gingival index, Plaque index, Adolescents, Nigeria

## Abstract

**Objectives:**

We describe the prevalence, and individual and familial risk indicators for dental caries and gingivitis among 10–19-year-old adolescents in Ile-Ife, South-West Nigeria.

**Methods:**

This cross-sectional study collected data through household surveys conducted between December 2018 and January 2019. Adolescents were recruited through multistage sampling. Oral health outcomes were caries, measured by the ‘Decayed, Missing due to caries, and Filled Teeth’ (DMFT) index, and gingivitis, measured by the Loe and Silness gingival index. Explanatory variables were individual (sex, age, oral health perception) and familial (socioeconomic status, birth rank, family size and parental living status) factors. Oral health behaviors (daily tooth-brushing, use of fluoridated toothpaste, consuming refined carbohydrates in-between meals, use of dental floss, dental service utilization in past 12 months, and smoking habits) were treated as confounders. Poisson regression models with robust estimation were constructed to determine associations between explanatory factors and oral health outcomes.

**Results:**

A total of 1472 adolescents were surveyed. Caries prevalence was 3.4%, with mean (standard deviation) DMFT of 0.06 (0.36) and plaque index of 0.84 (0.56). Only 128 (8.7%) adolescents brushed their teeth twice daily, 192 (16.1%) used dental floss daily, 14 (1.1%) utilized dental services in the last 12 months, and 508 (36.1%) consumed refined carbohydrates in-between meals less than once daily. The proportion of respondents who currently smoked cigarettes was 1.6%, and 91.7% of respondents used fluoridated toothpaste daily. The adjusted prevalence ratio of having caries increased by 18% for every additional age-year (APR: 1.18; 95% CI 1.004, 1.34). Additionally, participants with high socioeconomic status had significantly lower prevalence of caries compared to those with lower status (APR: 0.40; 95% CI 0.17, 0.91). Moderate/severe gingivitis was significantly associated with higher frequency of consuming refined carbohydrates in-between meals (APR: 2.33; 95% CI 1.36, 3.99) and higher plaque index scores (APR: 16.24; 95% CI 9.83, 26.82).

**Conclusion:**

Caries prevalence increased with increasing age and was higher among Nigerian adolescents with low socioeconomic status, while moderate/severe gingivitis was associated with frequent consumption of refined carbohydrates and higher plaque index score. While behavioral interventions may reduce the risk of gingivitis, structural interventions may be needed to reduce the risk for caries in this population.

## Background

Caries in the permanent teeth is the most prevalent oral health problem globally [1], and the prevalence of untreated caries in the permanent dentition peaks in adolescence [2]. Untreated caries has negative impact on quality of life [3] increasing the risk of tooth loss, with associated negative psychological [5, 6], social [6], nutritional [7], and physical [5] consequences. In addition, it is an adverse event often occurring at the physiologically and socially vulnerable life-period of adolescence, with potentially long-term health impact [4].

Adolescents from resource-limited settings are more likely to be significantly affected by chronic untreated caries. It can cause debilitating pain and infections, resulting in reduced quality of life and lost productivity from school and work [8]. Limited health infrastructure, low socioeconomic status and lack of health insurance in these settings increase the risk and impact of untreated caries, and poorer quality of life [9]. In sub-Saharan Africa, increasing urbanization and a concomitant rise in the consumption of refined carbohydrates has contributed to an increase in the prevalence of oral diseases [9].

Gingivitis is a form of periodontal disease that also affects quality of life [10], regardless of whether it is self-reported or clinically observed [11]. Severe periodontal disease was the 11th most prevalent condition in the world affecting about 11.2% of the world's population. The prevalence ranges from 20 to 50% [12, 13]. Though adolescents had the highest prevalence of no periodontal disease when compared with adults and older population, adolescents have higher prevalence of periodontal bleeding on probing and calculus when compared with adults and older population [14]. Caries is a risk factor for gingivitis, as it serves as a plaque retention factor through multiple pathways [15]. As such, the presence of these conditions is debilitating for affected patients, and in adolescence, could be regarded as opportunities for critical and timely health interventions.

Vulnerability to oral disease is high during adolescence, as it is a developmental period in which risk-taking behaviors often spike, contributing to poor oral health, among other things [16]. The transition period from childhood to adulthood is one in which personal values and self-sufficiency are typically established [17]. In this period, socialization is largely through peers and the media, and less so through parents [18]. Adolescents’ growing independence also means less parental/adult supervision for oral health and nutrition, and an increase in intake of highly sugar-containing products [19]; all factors associated with caries and periodontal diseases [15].

The available evidence on caries in Nigeria indicates that the prevalence in adolescents 10–16 years old varies by settlement type. Caries prevalence ranges from 5.9% in sub-urban Nigeria [20], to 12.2% in a rural population [21] and from 11.2% to 15.5% in urban populations [21–23]. Gingivitis however appears far more prevalent, with 95% of 11–16-year-old students in an urban secondary school showing evidence of periodontitis [24].

Known risk factors for caries are poor oral health perception [25] and poor oral health behaviors such as low frequency of tooth-brushing [26], non-use of fluoridated toothpaste [27], poor dental service utilization [25], high frequency of consumption of refined carbohydrates [28], and cigarette smoking [29, 30]. Sociodemographic variables such as sex (females are more at risk for caries than males) [31, 32] and age (the risk for caries increases with age) [33]; and familial factors such as low socioeconomic status [34], birth rank (last primogenitors) [35], large family size [35] and parental living status (absence of one or both parents) [36] can also be risk factors for caries. These factors may also increase the risk for periodontal diseases, which affect more than 10% of the global population [37].

It is important to obtain robust data for oral health interventions towards adolescent health and wellbeing as health planning can be challenging, especially in low-resource settings, because of poor availability of data to drive strategies and policy [38]. Nigeria is a low-resource country with a high burden of untreated dental caries [39]. For adolescents in particular, oral health services are inadequate in both availability and quality, as it is for adolescent health care in general [40]. Although there is some oral health data in Nigeria that may include the 10–19-year adolescent demographic, these data are often generated to reflect children under 10 or under 15 years of age. Some data are available on the epidemiology of caries in the 10–16-year age group [20–23, 41–43]; however, there is no data on caries and periodontal diseases in older adolescents 17–19-year-old, and limited data on periodontal diseases among 10–16-year-olds [24].

The aim of this study was to determine the prevalence of dental caries and gingivitis among 10–19-year-old adolescents resident in southwestern Nigeria, and to identify risk factors for these oral diseases in the study population.

## Methods

### Study setting, study population and study design

This was a cross-sectional study, where data were collected through a household survey conducted between December 2018 and January 2019. The study was conducted in Ife Central Local Government Area, a semi-urban community in Osun State, Southwestern Nigeria. Adolescents were eligible for the study if they were between 10 and 19 years old and living in the study setting. Adolescents who were critically ill and/or unable to respond independently to the survey were excluded from participation. Written individual informed consent, and parental informed consent with or without assent were obtained as prescribed by national guidelines (See Ethical Considerations). Recruitment of participants continued until study sample size was reached.

### Sample size and sampling technique

The minimum sample size was calculated with the formula proposed by Araoye. The formula is N = t^2 ^× p(1 − p)/m^2^, where N is the required sample size, t is the confidence level at 95% precision, p is the prevalence of caries for adolescents in the populace and m is margin of error at 5% [44]. With a caries prevalence of 13.9% among 12-year-olds in the study setting [17], a margin of error of 5%, and a confidence level of 95%, the minimum sample size determined was 1323 adolescents. This was increased to accommodate expected 10% loss due to non-response and/or refusal to participate with final sample size needed being 1455.

Adolescents were recruited with a multi-stage sampling technique. First, 70 of the 700 enumeration areas in Ife Central Local Government Area were sampled with the simple random technique. Next, in each selected enumeration area, every second household was enlisted for study participants’ recruitment. Finally, in each household, one adolescent who met the inclusion criteria was recruited for study participation. Where there was more than one eligible adolescent, a ballot was used to select the study participant who would be recruited for the study. The other adolescent(s) was/were also screened but their data were not collected for the study. Whenever a household declined to participate, the next eligible household was used as a substitute.

### Conceptual framework

The selection of explanatory variables was partly based on the conceptual model by Fisher-Owens et al. [45]. According to this model, the oral health of children may be affected by factors related to the child, family, and community. In the present study, we focused only on factors related to the child (individual factors) and family (familial factors).

### Demographic profile

Information on the age, sex, family composition/size, birth rank and socioeconomic status of the study participant was collected. Age was established as the adolescents’ age at their last birthday. Sex was determined as male or female. The birth rank of the study participant was determined as the birth position among his or her biological siblings. This was categorized into first/only child, and others [46]. The living arrangement of the adolescent’s household was also recorded as follows: child living with both parents, with mother only, with father only, with mother/father and step-parent or with a caregiver. For the regression analysis, living arrangement was dichotomized to “living with both parents” and “not living with both parents”.

### Socioeconomic status

Socioeconomic status was determined with an adapted version of the index developed by Olusanya et al. [47], which had been used for a previous study in the same setting [48]. This multiple-item index combines the mother’s level of education with the father’s educational level and occupation. The social class of the adolescent was determined by adding the score of the mother’s level of education to that of the father’s occupation. Each adolescent was allocated into social classes I–V (class I, upper class; class II, upper middle class; class III, middle class; class IV, lower middle class; class V, lower class). Where an adolescent had lost a parent, socioeconomic status was determined using the status of the living parent. Where an adolescent had lost both parents, socioeconomic status was determined using the status of the caregiver/guardian.

### Oral health perception

The process of assessing oral health perception for this study population has been described in detail in a prior publication [49]. Briefly, respondents were asked to react to eight statements about aspects of caries diagnosis and prevention on a five-point Likert scale ranging from “strongly agree” to “agree”, “neutral”, “disagree”, and “strongly disagree”. The statements ranged from ‘*Use of fluoride-containing toothpaste is an effective, safe, and efficient way to prevent holes from forming on the teeth’* to ‘*It is important to visit the dental clinic regularly as a measure for preventing holes from forming on the teeth’*. The responses were then scored from one to five, with “strongly agree” scoring 5 and “strongly disagree” scoring 1. Therefore, the total minimum and maximum scores attainable were 8 and 40, respectively. [49].

### Tooth-brushing

Respondents were also asked to indicate the frequency of tooth-brushing using the following response options—‘*irregularly or never’*, *‘once a week’*, *‘a few (2–3) times a week’, ‘once a day’*, and *‘more than once a day’.* Respondents who chose the options *‘irregularly or never, once a week, a few (2–3) times a week’;* or *‘once a day’* were classified as not having undertaken preventive dental care [50].

### Use of fluoridated toothpaste

Respondents were asked to indicate the frequency of their use of fluoridated toothpaste when tooth brushing using the following options—*‘Always’, ‘quite often’, ‘seldom’, ‘not at all’*. Respondents who answered *‘quite often’, ‘seldom’,* or *‘not at all’* were classified as not having undertaken preventive dental care [50].

### Consumption of refined carbohydrates in-between meals

Respondents were asked to indicate the frequency of consuming refined carbohydrates in the form of snacks or drinks in-between meals, using the following options—*‘About 3 times a day or more, about twice a day, about once a day, occasionally; not every day, rarely or never eat between meals’*. Those who chose the options *‘About 3 times a day or more’, ‘about twice a day’,* or *‘about once a day’*, were classified as not having undertaken preventive dental care [50].

### Use of dental floss

Respondents were asked to indicate how often they used dental floss by choosing from the following options—*‘Not at all, occasionally, a few (2–3) times a week, once in a day, more than one time in a day’*. Respondents who selected *‘Not at all’, ‘occasionally’,* or *‘a few (2–3) times a week’* were classified as not having undertaken preventive dental care [50].

### Dental service utilization

Respondents were to indicate the time of their last dental check-up as follows—*‘within the last 6 months, more than 6 months to one year ago, more than 1–2 years ago, more than 2–5 years ago, more than 5 years, never, do not remember’*. Attending a dental check-up within the last one year was defined as preventive care use. Respondents who chose the options ‘*more than 1–2 years ago’, ‘more than 2–5 years ago’, ‘more than 5 years ago’, ‘never’,* or ‘*do not remember’* were classified as not having undertaken preventive dental care [50].

### Smoking habits

The questionnaire separately requested information on respondents’ cigarette smoking habits. There were response options—*‘No, never, No—I used to, but I quit, Yes, once a month or less, Yes—a few times (2–3) a month, Yes—a few times (2–3) a week, Yes-once a day or more’*. Responses were categorized into current smokers, former smoking habits, and non-smoking. All those who chose options *‘Yes—once a month or less’, ‘Yes—a few times (2–3) a month’, ‘Yes-a few times (2–3) a week’,* or *‘Yes-once a day or more’* were classified as current smokers [50].

### Oral hygiene

Each participant was examined sitting, under natural light, and with sterile dental mirrors by trained dentists. The teeth were examined wet. Plaque index [51] was used to determine oral hygiene status. The plaque index score was based on six numerical determinations representing the amount of debris found on the surfaces of index permanent teeth 12, 16, 24, 32, 36, and 44. The mesial, distal, buccal, and lingual gingival areas of the index teeth are scored from 0 (no plaques) to 3 (abundance of soft matter within the gingival pocket and/or on the tooth and gingival margin). The mean score for each tooth was obtained and the mean score for the individual was determined by adding the indices for each tooth and dividing by the number of teeth examined.

### Gingival health

The presence and severity of gingivitis was evaluated with the gingival index [51]. Changes in the gingiva in relation to the appropriate six index teeth (16, 12, 24, 36, 32 and 44) in the permanent dentition were assessed. Four areas of each index tooth were scored, and the scores were summed and divided by four to give the gingival index for each tooth. The gingival index of each participant was obtained by adding the values of all index teeth and dividing by six. Gingivitis was classified as none (healthy gingiva), or mild, moderate, or severe gingivitis, with values of 0, 0.1–1.0, 1.1–2.0, and 2.1–3.0, respectively. Gingivitis was dichotomized into ‘healthy gingiva/mild gingivitis’ versus ‘moderate/severe gingivitis’ for the logistic regression analysis [52].

### Caries

Each adolescent was examined sitting, under natural light, and with sterile dental mirrors by trained dentists. The teeth were cleaned of debris and dried using a sterile gauze. Caries examination was conducted after completing examinations for oral hygiene and gingival health status. The caries status of each permanent tooth was assessed with the decayed (D), missing (M), and filled (F) teeth (DMFT) index, following World Health Organization criteria [53]. The DMFT index was obtained by adding the D, M and F scores respectively. Caries status was further categorized into caries present (DMFT > 0) or absent (DMFT = 0).

### Standardization of examiners

Clinical investigators were three qualified dentists undergoing postgraduate residency training as pedodontists, who were calibrated on the study protocol and the clinical examination procedures. Training was conducted by a consultant pedodontist with over 20 years of practice experience. This was followed by practice on 10 adolescent patients: each of the three clinicians examined and scored the adolescents for oral hygiene and caries status as prescribed in the study protocol. Results were subjected to a Cohen’s weighted kappa score analysis to determine inter-examiner reliability. Each clinician’s score was compared with that of the consultant pedodontist. The inter-examiner Cohen’s weighted kappa scores for the three dentists were all greater than 0.95.

### Data analysis

Descriptive analyses were conducted to determine the proportion of adolescents with each individual factor: sociodemographic variables (age, sex), ‘perception of oral health’ score, oral health behaviors (tooth-brushing, use of fluoridated toothpaste, frequency of consumption of refined carbohydrates in-between meals, flossing, utilization of dental services and smoking) plaque accumulation, and familial factors (socioeconomic status, birth rank, family size and parental living status). Bivariate analyses using *t* test and chi square tests were conducted to determine associations between the explanatory and outcome variables (the presence of caries in permanent teeth and the presence of moderate/severe gingivitis).

Poisson regression models were constructed to determine the association between risk indicators and the two oral health clinical outcome variables (caries and gingivitis). For each outcome variable, three models were constructed: Model 1 including individual factors, Model 2 including familial factors, and Model 3 including individual and familial factors. The estimated coefficients (expressed as adjusted prevalence ratios (APR) and their 95% confidence intervals) were calculated, as well as log likelihood as measure of model fit. Statistical analysis was conducted using SPSS for Windows version 23.0 (IBM Corp., Armonk, NY, USA). Statistical significance was inferred at *p* ≤ 0.05.

### Ethical considerations

Ethical approval was obtained from the Ethics and Research Committee of the Institute of Public Health, Obafemi Awolowo University, Ile-Ife, Nigeria. Approval for study implementation was obtained from the Local Government Authority prior to commencement. Written parental consent was obtained for participants aged 10–11 years. Written parental consent and written participant assent were obtained for those aged 12–13-years. Written consent was obtained from study participants aged 14–19 years in line with guidance from the Federal Ministry of Health [54]. Efforts were made to ensure confidentiality by ensuring anonymized data collection was done privately using an electronic data platform. Study participants’ discomfort with the personal nature of questions was limited by ensuring field workers were trained on how to ask sensitive questions and on clarifying non-verbal cues observed during the interviews. No compensation was paid to adolescents for study participation. Each participant received a gift of toothpaste of a value less than $1.00.

## Results

**Fig. 1:**
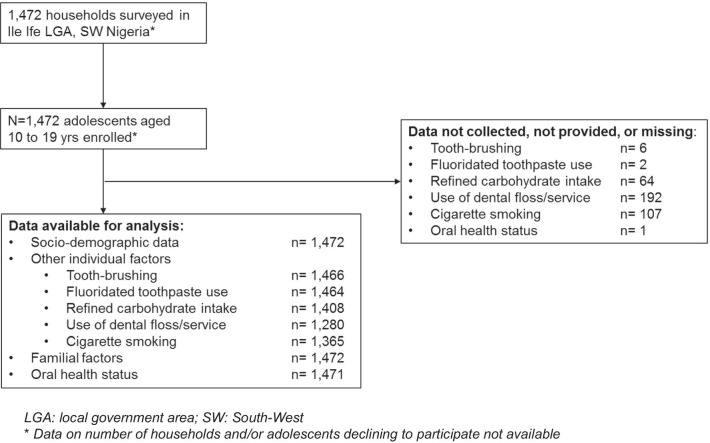
Study Flow Chart. *LGA* local government area, *SW* South-West. *Data on number of households and/or adolescents declining to participate not available

A total of 1472 adolescents (11.3% above minimum sample size) were enrolled from an identical number of households for the survey. A flowchart (Fig. 1) was developed using the STROBE flow chart as a guide [55].

Table 1 shows the profile of the study participants. The prevalence of caries was 3.4%, and the prevalence of mild, moderate and severe gingivitis was 57.2%. 7.8% and 0.7% respectively. The mean (standard deviation) DMFT was 0.06 (0.36) and plaque index was 0.84 (0.56). The proportion of adolescents that undertook caries and gingivitis prevention practices was low. Only 8.7% of adolescents brushed their teeth twice daily, 16.1% used dental floss daily, 1.1% utilized dental services in the last 12 months, and 36.1% consumed refined carbohydrates in-between-meals less than once a day. The prevalence of current smokers were however, very low—1.6%—and the use of fluoridated toothpaste was very high at91.7%.

**Table 1:** Individual and familial factors, and oral health status of study participants (n = 1472)

Variables	Total
	N (%)
*Individual factors*	
Male	846 (57.5)
Female	626 (42.5)
Perception of oral health: mean (SD)	19.04 (8.58)
*Individual factors: oral health behaviors*	
Tooth-brushing	
Once a day or less	1338 (91.3)
2 times a day or more	128 (8.7)
Use of fluoridated toothpaste	
Always	1343 (91.7)
Not always	121 (8.3)
Consumption of refined carbohydrates in-between-meals	
Frequent	900 (63.9)
Non-frequent	508 (36.1)
Use of dental floss	
Daily	192 (16.1)
Not daily	1002 (83.9)
Utilized dental services in the last 12 months	
Yes	14 (1.1)
No	1266 (98.9)
*Cigarette smoker*	
Current	22 (1.6)
Former	8 (0.6)
Non-smoker	1365 (97.8)
*Familial factors*	
Birth rank	
First/only child	570 (38.7)
Second or more child	902 (61.3)
Family size	
6 or less	1184 (80.4)
More than 6	288 (19.6)
Living arrangement	
Living with both parents	1199 (81.5)
Not living with both parents	273 (18.5)
Socio-economic status	
High	490 (33.3)
Middle	509 (34.6)
Low	473 (32.1)
*Oral health status*	
Prevalence of caries in permanent teeth	50 (3.4)
Mean DMFT (SD)	0.06 (0.36)
Mean plaque index (SD)	0.84 (0.56)
Normal gingival condition	505 (34.3)
Mild gingival inflammation	841 (57.2)
Moderate gingival inflammation	115 (7.8)
Severe gingival inflammation	10 (0.7)

*DMFT *decay, missing, filled teeth, *S*D standard deviation

Table 2 highlights individual and familial factors associated with the presence of caries in the permanent dentition using a bivariate analysis. The only factor significantly associated with the presence of caries in permanent teeth was age (*p* = 0.001).

**Table 2:** Association between the presence of caries in permanent dentition and individual and familial factors in bivariate analysis

Variables	Caries absent	Caries present	*P* value
	N (%)	N (%)	
*Sex*			
Male	815 (57.3)	31 (62.0)	0.51
Female	607 (42.7)	19 (38.0)	
*Age*			
Mean (SD)	14.51 (2.59)	15.72 (2.65)	0.001^a^
Perception of oral health	19.04 (9.13)	19.04 (8.56)	0.99
*Tooth-brushing*			
1 time a day or less	1292 (91.2)	46 (92.0)	1.00
Equal to or greater than 2 times a day or more	124 (8.8)	4 (8.0)	
*Use of fluoridated toothpaste*			
Always	1297 (91.7)	46 (92.0)	1.00
Not always	117 (8.3)	4 (8.0)	
*Consumption of refined carbohydrates in-between-meals*			
Frequent sugar consumption	868 (63.9)	32 (64.0)	0.99
Non-frequent sugar consumption	490 (36.1)	18 (36.0)	
*Daily use of dental floss*			
Yes	184 (16.0)	8 (19.5)	0.54
No	969 (84.0)	33 (80.5)	
*Dental service utilization in the last 12 months*			
Yes	14 (1.1)	0 (0)	1.00
No	1220 (98.9)	46 (100)	
*Cigarette smoking*			
Current smoker	20 (1.5)	2 (4.0)	0.33
Former smoker	8 (0.6)	0 (0)	
Non-smoker	1317 (97.9)	48 (96.0)	
*Plaque index*			
Mean (SD)	0.81 (0.52)	0.84 (0.56)	0.74
Gingival index			
Mean (SD)	0.53 (0.48)	0.54 (0.53)	0.93
*Birth position*			
First/only child	553 (38.9)	17 (34.0)	0.49
Second or more child	869 (61.1)	33 (66.0)	
*Family size*			
6 or less	1143 (80.4)	41 (82.0)	0.78
More than 6	279 (19.6)	9 (18.0)	
*Living arrangement*			
Living with both parents	1157 (81.4)	42 (84.0)	0.64
Not living with both parents	265 (18.6)	8 (16.0)	
*Socioeconomic status*			
High	476 (33.5)	14 (28.0)	0.61
Middle	492 (34.6)	17 (34.0)	
Low	454 (31.9)	19 (38.0)	

^a^Statistically significant at *p* < 0.05

In the multivariable regression analysis highlighted in Table 3, age was the only individual factor associated with the presence of caries in permanent dentition (Model 1). In Model 2, there were no significant associations between the presence of caries in permanent teeth and any of the familial factors. In the full model (Model 3), the only significant associations were with age and socioeconomic status. Every one year increase in age was significantly associated with higher prevalence of caries when compared with younger adolescent (APR: 1.18; 95% CI 1.04, 1.34). Also, participants from higher socioeconomic status had significantly lower prevalence of caries than those with low status (APR: 0.40; 95% CI 0.17, 0.91). Model 3 had the best fit (lowest LL among the 3 models = 339.29). However, the familial factors in Model 2 significantly explained the presence of caries in the permanent dentition (*p* = 0.03).

**Table 3:** Association between the presence of caries in permanent dentition and individual and familial factors in regression analysis

Factors	APR (95% CI)		
	Model 1	Model 2	Model 3
Male versus female	1.01 (0.53, 1.92)	1.15 (0.64, 2.06)	1.01 (0.53, 1.91)
Age	1.14 (1.01, 1.29)^a^	1.23 (1.10, 1.39)^a^	1.18 (1.04, 1.34)^a^
Perception of oral health	1.01 (0.97, 1.05)	–	1.00 (0.96, 1.04)
Twice or more daily tooth brushing a day versus tooth-brushing less than twice daily	0.93 (0.32, 2.70)	–	0.84 (0.29, 2.45)
Always using fluoridated toothpaste versus not always using fluoridated toothpaste	0.91 (0.27, 3.09)	–	0.87 (0.25, 3.00)
Frequent versus non-frequent consumption of refined carbohydrates in-between-meals	1.20 (0.60, 2.40)	–	1.25 (0.62, 2.53)
Daily use of dental floss versus non-daily use of dental floss	0.72 (0.32, 1.62)	–	0.71 (0.31, 1.63)
Plaque index	0.91 (0.51, 1.61)	–	0.92 (0.52, 1.65)
First/only child versus second or more birth rank	–	1.30 (0.71, 2.40)	1.23 (0.63, 2.41)
Less than 6 versus 6 or more family size	–	1.26 (0.60, 2.67)	1.48 (0.59, 3.69)
Living with both parents versus not living with both parents	–	0.70 (0.32, 1.53)	0.42 (0.14, 1.24)
High versus low socioeconomic status	–	0.49 (0.24, 1.02)	0.40 (0.17, 0.91)^a^
Middle versus low socioeconomic status	–	0.74 (0.38, 1.45)	0.57 (0.27, 1.23)
LL	347.47	420.88	339.29

*APR* adjusted prevalence ratio, *CI* confidence interval

^a^Statistically significant at *p* < 0.05

Model 1 includes individual factors

Model 2 includes familial factors

Model 3 includes individual and familial factors

Adding the presence of moderate/severe gingivitis to Model 3, APR = 1.16, 95% CI 0.31, 4.33

Table 4 highlights the factors associated with gingivitis. The presence of moderate/severe gingivitis was significantly associated with frequent consumption of refined carbohydrates in-between meals (*p* = 0.002), and plaque index (*p* < 0.0001). Tooth-brushing frequency was not associated with gingivitis.

**Table 4:** Association between the presence of moderate/severe gingivitis and individual and familial factors in bivariate analysis (N = 1472)

Variables	Normal and mild gingivitis	Moderate and severe	*P* value
	N (%)	N (%)	
*Sex*			
Male	769 (57.1)	77 (61.6)	0.33
Female	577 (42.9)	48 (38.4)	
*Age*			
Mean (SD)	14.53 (2.61)	14.74 (2.57)	0.41
*Perception of oral health*			
Mean (SD)	19.07 (8.63)	18.70 (8.00)	0.65
*Tooth brushing*			
Once a day or less	1227 (91.6)	110 (88.0)	0.18
Twice a day or more	113 (8.4)	15 (12.0)	
*Use of fluoridated toothpaste*			
Always	1223 (91.3)	119 (96.0)	0.07
Not always	116 (8.7)	5 (4.0)	
*Consumption of refined carbohydrates in-between-meals*			
Frequent sugar consumption	840 (652)	60 (50.8)	0.002^a^
Non-frequent sugar consumption	449 (34.8)	58 (49.2)	
*Daily use of dental floss*			
Yes	922 (84.2)	79 (80.6)	0.35
No	173 (15.8)	19 (19.4)	
*Dental service utilization in the last 12 months*			
Yes	11 (0.9)	3 (2.6)	0.10
No	1153 (99.1)	112 (97.4)	
*Cigarette smoking*			
Current	20 (1.6)	2 (1.9)	0.70
Former	8 (0.6)	0 (0.0)	
Non-smoker	1259 (97.8)	105 (98.1)	
*Plaque index*			
Mean (SD)	1.57 (0.54)	0.77 (0.51)	< 0.0001^a^
*Birth position*			
First/only child	515 (38.3)	54 (43.2)	0.28
Second or more child	831 (61.7)	71 (56.8)	
*Family size*			
Less than 6	258 (19.2)	30 (24.0)	0.19
6 or more	1088 (80.8)	95 (76.0)	
*Living arrangement*			
Living with both parents	1103 (81.9)	96 (76.8)	0.16
Not living with both parents	243 (18.1)	29 (23.2)	
*Socioeconomic status*			
High	448 (33.3)	42 (33.6)	0.65
Middle	470 (34.9)	39 (31.2)	
Low	428 (31.8)	44 (35.2)	

^a^Statistically significant at *p* < 0.05

In the multivariable regression analyses highlighted in Table 5, Model 1 showed that the frequent consumption of refined carbohydrates in-between meals and plaque index were significantly associated with the presence of moderate/severe gingivitis. None of the familial factors in Model 2 were significantly associated with the presence of moderate/severe gingivitis. In Model 3, which was fully adjusted for individual and familial factors, the presence of moderate/severe gingivitis was significantly associated with frequent consumption of refined carbohydrates in-between meals (APR; 2.33; 95% CI:1.36, 3.99) and higher plaque index scores (APR: 16.24; 95% CI 9.83, 26.82). Compared to participants who had low frequency of consumption of refined carbohydrates in-between meals, those who did so more frequently had more than two-fold higher prevalence of moderate/severe gingivitis. Also, those with one score higher plaque index had 16 times higher prevalence of moderate/severe gingivitis. Model 3 had the best fit, indicated by the lowest LL among the three models (452.67).

**Table 5:** Association between the presence of moderate/severe gingivitis and personal and familial factors in regression analysis

Factors	APR (95% CI)		
	Model 1	Model 2	Model 3
Male versus female	1.21 (0.73, 2.02)	1.19 (0.82, 1.74)	1.21 (0.72, 2.03)
Age	0.99 (0.90, 1.10)	1.03 (0.95, 1.10)	1.00 (0.90, 1.11)
Oral health perception	1.01 (0.98, 1.05)	–	1.01 (0.98, 1.05)
Twice or more daily tooth- brushing versus less than twice a day	0.92 (0.42, 2.01)	–	0.95 (0.43, 2.10)
Always using fluoridated toothpaste versus not always using fluoridated toothpaste	0.47 (0.15, 1.43)	–	0.44 (0.14, 1.38)
Frequent versus non-frequent consumption of refined carbohydrates in-between meals	2.29 (1.35, 3.88)^a^	–	2.33 (1.36, 3.99)^a^
Daily use of dental floss versus non-daily use of dental floss	0.97 (0.50, 1.90)	–	0.99 (0.50, 1.96)
Plaque index	16.55 (10.13, 27.04)^a^	–	16.24 (9.83, 26.82)^a^
First/only child versus second or more birth rank	–	0.74 (0.51, 1.09)	0.81 (0.48, 1.39)
Less than 6 versus 6 or more family size	–	0.70 (0.45, 1.10)	0.68 (0.36, 1.27)
Living with both parents versus not living with both parents	–	1.36 (0.87, 2.12)	0.77 (0.40, 1.48)
High versus low socioeconomic status	–	0.92 (0.58, 1.46)	0.92 (0.47, 1.77)
Middle versus low socioeconomic status	–	0.80 (0.51, 1.26)	0.92 (0.50, 1.70)
LL	455.45	847.49	452.67

*APR* adjusted prevalence ratio, *CI* confidence interval

^a^Statistically significant at *p* < 0.05

Model 1 includes individual factors, Model 2 includes familial factors, Model 3 includes individual and familial factors

## Discussion

Our study presents data on the prevalence of caries and gingivitis among adolescents in semi-urban, South-Western Nigeria. It showed that although the proportion of adolescents that practiced caries prevention behaviors were low, the prevalence and severity of caries was unexpectedly low. The risk factors significant for caries were age and socio-economic status, with the risk of caries being higher in older adolescents and among those with low socioeconomic status. Most adolescents had mild gingivitis. Risk factors for moderate/severe gingivitis differed from that of caries: the severity of gingivitis was higher among adolescents who frequently consumed refined carbohydrates in-between meals and for those with higher plaque index scores.

One of the strengths of this study was the collection of data using a household survey and the sample size, which enabled adequate power for the determination of the prevalence of caries and gingivitis for the target study population. Also, the study was able to determine the proportion of the population who had preventive oral health behaviors. This data is therefore able to support the generation of country-level prevalence data on risk factors for poor oral health among 10–19-year-olds in Nigeria. However, the cross-sectional nature of the study makes it difficult to determine a causal relationship between the outcomes and explanatory variables, or the direction of the relationships between the variables. The prevalence of caries would have been underestimated since caries experience was only determined clinically and not radiographically; and the clinical evaluation of caries was based on presence of cavitated and not demineralized caries. In addition, participants may have exaggerated reporting of tooth-brushing and dental service utilization due to social desirability and recall bias, respectively. The extremely low self-reported frequency of dental service utilization reduces the risk of social desirability bias in the reporting of this variable. Despite these limitations, the study findings provide useful data on adolescent oral health that may inform the design of oral health programs for the target population.

Our findings indicate that the prevalence of caries is low among adolescents in this Nigerian cohort. The prevalence of caries in the permanent dentition reported in this study is lower than the 5.9% reported from a school-based population of 6–16-year-old pupils in the same study setting [29], and in high contrast to the prevalence of 58% reported among 12–19-year-olds in the United States of America in 2011–2012 [56], 66.0% for 11–19-year-old Ugandans [57], and 95.5% among 10–19-year-old Romanians [58]. There are currently no studies identifying protective factors for caries in the environment, although it is possible that the use of fluoridated toothpaste by a large percentage of the population may be a plausible protective factor. A prior study indicated that the caries experience was higher for those who use non-fluoridated toothpaste in Nigeria than those who used fluoridated toothpaste [59]. It is also possible that the consumption of starchy staple foods and fresh fruits which are found and eaten in abundance in the study environment, may be associated with the low level of dental caries observed [60] though no studies have been conducted to explore and possible association. These postulations need to be investigated further.

The observed higher caries prevalence with older age in this study has been reported in prior studies [59, 61], and this had been ascribed to longer exposure of the teeth to cariogenic insults in the oral environment [62]. Recent evidence suggests that exposure to environmental insults such as air pollutants like nitrous oxide, may also be a risk factor for caries [63].

In this study, we found that high frequency of consumption of refined carbohydrates in-between meals was a significant risk indicator for gingivitis. Available evidence suggests that high sugar consumption is not only a risk factor for caries but also for periodontal disease [64]. Sugar may lead to increased inflammation and oxidative stress that in turn trigger a hyper-inflammatory state [65, 66], resulting in increased bleeding at the dental probing, probing depth, and attachment level [67–69].

The results of this study are very important: first, the frequency of consumption of refined carbohydrates in-between meals is a risk indicator for gingivitis for this adolescent population, and not for caries, as reported by prior studies conducted in the study setting and in other countries [29, 70]. Second, the frequency of tooth-brushing was not associated with gingivitis in this study, which contradicts findings from prior reports in other settings [71]. It is important to determine modifiers of the relationship between caries, gingivitis and long-established risk indicators in other populations that may not so act in this study population.

The high prevalence of having moderate/severe gingivitis being associated with higher plaque index score is not surprising [72]. Gingivitis resulting from plaque accumulation is a consequence of poor oral hygiene [73] and is associated with poor quality of tooth-brushing, rather than the low frequency of daily tooth-brushing [74]. This study did not assess the quality of tooth-brushing and is therefore unable to provide evidence to support this postulation. Further research will be needed to understand the impact of both the quality and frequency of tooth brushing f on the risk for gingivitis among adolescents. Studies aiming determine how to reduce the risk for gingivitis are important to pursue, because potentially cheap and effective gingivitis risk-reducing interventions can ultimately reduce long-term risks for developing cardiovascular and endocrine disorders in the future [75].

Although the prevalence of cigarette smoking was low in the study population, prevention efforts need to be instituted, strengthened and/or sustained to promote practices that have kept this prevalence low. Studies also need to be conducted to minimize the use of alternative oral tobacco products, which are also risk factors for periodontal diseases but were not assessed in the present study.

The study findings provide data on the prevalence of caries and gingivitis for adolescents howbeit for a sub-urban population in Nigeria. It however starts the process of supporting the goal of addressing the oral health of adolescents in Nigeria as indicated in the 2020 National oral health policy where it noted a limitation in developing contextual evidence-informed policy for oral health in adolescents due to the limited accessibility to relevant adolescent oral health data [76]. The data generated through this study may also help in developing estimates for use in modelling studies for other countries in West Africa or Africa where data on the epidemiological profile of adolescents’ oral health is sparse. Our study also sheds light on factors associated with two oral diseases of low prevalence in this group of adolescents thus providing evidence that may apply to similar groups with low disease prevalence. Thisstudy contrast with most previous studies conducted among populations with higher disease levels.

## Conclusion

The study indicates that the prevalence of caries increases with age and may be higher among adolescents with low socioeconomic status. Furthermore, the prevalence of moderate/severe gingivitis may be higher for adolescents who frequently consume refined carbohydrates in-between meals, and among those with high plaque index scores. While behavioral interventions may reduce the risk of gingivitis, macro-level structural interventions that reduce the possibility for socio-economic status to be a determinant of health, such as poverty alleviation, may be needed to reduce the risk for caries among adolescents in South-Western Nigeria. Future studies are needed to assess whether the study findings are consistent for adolescents in other parts of Nigeria and in other African countries, and if our findings are comparable to those for adolescents from other countries with similar and different socioeconomic and demographic profiles.

## Data Availability

All data generated for this study are presented in the manuscript. Patient-level data can however be accessible on reasonable request from the corresponding author, Morenike Oluwatoyin Folayan.
